# Supplementary material for: Extracting TNFi switching reasons and trajectories from real-world data using large language models
Source: JAMIA Open. 2025 Nov 11;8(6):ooaf132. doi: 10.1093/jamiaopen/ooaf132 (PMC12605798; doi:10.1093/jamiaopen/ooaf132)
Supplement: ooaf132_Supplementary_Data [file ooaf132_supplementary_data.zip › TNF switch review UCSF.docx]

**Response to the reviewers**

**Reviewer 1**

Comments to the Author

This manuscript presents a compelling and methodologically rigorous study evaluating the use of large language models (LLMs), including GPT-4 and several open-source alternatives, to extract tumor necrosis factor inhibitor (TNFi) switching patterns and reasons from clinical notes. Using de-identified EHR data from UCSF spanning 2012–2023, the authors demonstrate that LLMs can automate chart review and outperform structured data in capturing nuanced clinical rationale for treatment changes.

The study has numerous strengths:

Innovative Use of LLMs: The study leverages GPT-4 and compares it with 13 open-source models, showcasing a robust benchmarking framework. The use of zero-shot prompting and multiple prompt designs adds depth to the evaluation. It makes a strong case for the use of LLMs in data abstraction and annotation at a large scale.

Comprehensive Dataset: The cohort includes 9,187 patients and 3,104 TNFi switches, providing a rich dataset for analysis.

**Author’s Response: Thank you for the support of our work.**

There are however a few areas that do need additional clarification:

1. Clarification Needed on Gold Standard Annotations:

While the manuscript references the use of gold-standard annotations for evaluating model performance (e.g., microF1 scores against expert-labeled data), it remains unclear how many clinical notes were manually reviewed to establish this gold standard. Specifically, the authors mention that a physician annotated data according to guidelines (referenced in Figure S1), but do not state whether this applied to the entire dataset or only a validation subset. Additionally, the distinction between silver-standard labels (derived from medication order data) and gold-standard annotations is not consistently delineated throughout the manuscript. Clarifying the scope and scale of manual chart review would strengthen the validity of the reported performance metrics and help readers better interpret the reliability of the model comparisons.

**Author’s Response: We thank the reviewer for this clarification. Gold-standard annotations were established through detailed manual chart review of a validation subset. Specifically, a physician reviewed 146 clinical notes (approximately 5% of the 2,112 TNFi switches within the total of 3,204 switch events). These annotations were performed according to the guidelines outlined in Figure S1. In contrast, silver-standard labels were derived from structured medication order data, which can be used to confirm switch events (for example prescriptions for new drugs) but do not capture the full clinical context, including the reason for the switch. The gold-standard chart review therefore represents the most reliable reference for model evaluation, as it incorporates clinical detail not available in structured data. We add the following information to the manuscript to clarify these details:**

**Page 7 lines 130-134:** *“Model performance was assessed against silver-standard labels from associated medication order information as well as gold-standard annotations provided by a physician (MB) according to annotation guidelines on 146 clinical notes representing approximately 5% of 2.12 TNFi switch patients (Figure S1) and microF1 scores were assessed.”*

2. Demographics table would benefit from a statistical comparison of the two groups.

**Author's response: We agree with the reviewer, and added p-value to Table 1 as a measure of significance of differences. We also added the following to our methods section to reflect this change:**

**Page 10 lines 188-190:** “Statistical testing was performed using chi-square tests for categorical variables and two-sided t-tests for continuous values. A p-value of less than 0.05 was considered statistically significant.”

3. Only the first clinical note per encounter was used, which may omit relevant context. A sensitivity analysis using multiple notes could be helpful in mitigating any possible selection bias.

**Author’s response: We apologize for the confusion surrounding this issue. The note used for this study was the last available note for the encounter. We intentionally made this choice since based on our prior experience, the last note is the final and most up to date version of the note (and captures any potential post-hoc edits). After careful consideration, we decided not to conduct additional analyses using previous versions of the note, since we note that the content is generally very similar (other than minor edits) and therefore, this will add a high degree of redundancy which will increase computational cost (and monetary cost with pay-per-use LLMs) without increasing available information. In addition, there may be slight contradictions that may confuse the model, as the last note may include corrections that are not present in earlier versions. We clarify our methods and limitations in the discussion to reflect this information. Additionally, we include a table in our supplemental data (Table S10) describing the notes in more detail.**

**Page 6 lines 97-99:** “*In addition, for encounters with multiple notes, only the last note was used, as it is the most up to date version available.”*

4. A comment clarifying why the dose changes and transitions to non-TNFi therapies were not captured would be helpful.

**Author’s Response: We thank the reviewer for this comment. In the present analysis, our primary focus was on TNFi treatment patterns as proof-of-concept. Therefore, dose modifications and transitions to non-TNFi therapies were not captured. As noted in the Discussion, given the good performance of our proposed method, future (currently ongoing) work will specifically examine all medication transitions, including non-biological DMARDs and other biologic therapies, to provide a more comprehensive understanding of treatment trajectories.**

**Page 18 lines 316-318** *“Another limitation is that our study did not explore disease-specific switching patterns in depth, nor did it account for changes to or from medications other than TNFi. Our future work will explore this possibility for a broader scope of medications and diseases.”*

5. Data Availability: The manuscript states that data are not available. Providing synthetic examples or a limited dataset (since it is deidentified) could enhance reproducibility. Also considering a code availability with a few examples of data could enhance adoption of this more widely.

**Author’s Response: Unfortunately, while we agree with the reviewer, we are not authorized by our institution to publicly disclose this clinical data at large. To mitigate this limitation, we have provided a de-identified validation set that includes the reasons extracted with GPT-4, along with both the gold-standard expert labels and the silver-standard labels from medication orders (Table S3). All of the code to reproduce our methods is openly available on GitHub and we’ve included example configurations to make it easier for others to adopt. We hope this will increase reproducibility and future studies at other locations.**

**Page 24 Lines 457-459:**  ***Data Availability Statement:*** *Data available: No . Code availability: All code used to replicate our methods is available at* [*https://github.com/BMiao10/TNFi-switching/tree/main/configs*](https://github.com/BMiao10/TNFi-switching/tree/main/configs)

Minor comments

1. Clarify whether the “Reasons provided” prompt was used uniformly across all models or tailored per model.

**Author’s Response: We confirm that a uniform ‘Reasons provided’ prompt was implemented across all models. Only template tags were tailored to individual models (using the “tokenizer.apply_chat_template” function built into each model), while the reasons remained consistent.**

Page 8 Lines 150-151: “The prompt was applied uniformly across all models, with only model-specific template tags varied.”

2. A few grammatical errors in the supplementary file would need to be corrected.

**Author’s Response: We thank the reviewer for their comment and have corrected the grammatical inconsistencies in the Supplementary section.**

**Reviewer 2**

Comments to the Author

This is a well motivated application of LLMs that demonstrates promise in idenitfiying information about why a patient switched TNFi therapies. Most valdiated clinical NLP tools have focused on concept extraction, which would be helpful towards indentifying reasons, but would be a non-trivial extension to extract resons for a change in treatment. It is also great to see the approach achieve good accuracy, per the F1 measure, using GPT-4. Also, that the findings reveal that lack of efficacy, followed by adverse events, and insurance or cost considerations are among the most frequently documented reasons for TNFi switching -- and condistant with prior literature indicating that treatment failure and immunogenicity drive many TNFi switches.

I have three comments that I feel would be helpful to clarify or further detail, as space allows.

#1 An explanation of why you only used the first note of every encounter. I suspect it was due to imporve accuracy, but it's not clear. What is the impact of avoiding consequent notes for precision and recall? Or what is for an altogether different reason?

**Author’s response: The reviewer is right, and we apologize for the confusion surrounding this issue. Please refer to our reply to Reviewer 1, who had the same comment.**

#2 How did you develop your prompt? Was it a subject matter expert? Did you experiment with different prompt engineering techniques? For example, did you tyr including some labeled examples in the prompt? If no, that would be good to know. If yes, lessons learned through the process of prompt enginnering would lbe great to share.

**Author’s response: We thank the reviewer for this question. Prompts were developed in collaboration with clinical experts to ensure clinical relevance. and with the use of the prompt development set for validation. The resulting prompts are provided in Table S2. The selected prompts were applied uniformly across all models, with only model-specific template tags varied to maintain comparability.**

**Methods section Page 13 lines 205-209: “**Prompts were developed in collaboration with clinical experts to ensure clinical relevance. and with the use of the prompt development set for validation. The resulting prompts are provided in Table S2. The selected prompts were applied uniformly across all models, with only model-specific template tags varied to maintain comparability.**”**

Laslty, I noticed that for consistency purposes, encounters where multiple TNFi were ordered on the same date. I was wondering why this would happen and how these patients might be different (if they even are) than other patients that are candidates for TNFi.

**Author’s response: We thank the reviewer for this observation. Encounters with multiple TNFi orders on the same date are unusual, and can happen for several reasons. For example, if the clinician is unsure about which medication will be covered by insurance. While these are excluded, we want to make it clear that this switch is still captured by our algorithm (for example, patient is on Humira in Encounter A, prescriptions for Humira and Enbrel are entered for Encounter B, and Encounter B is excluded, patient is on Enbrel on Encounter C -> A switch is detected between Encounters A and C from Humira to Enbrel). We did not explore this anomaly in depth, as it represents a small portion of the dataset (n = 6,600; 3.4%). We do not expect these cases to substantially affect the overall findings. However, we include a mention of this issue in our limitations section:**

**Discussion section Page 16 lines 312-315: *“****An additional limitation of our approach is that we did not study the impact of patients with multiple TNFi orders in the same encounter. However, this event is uncommon (n=x, y%) and we do not expect these cases to substantially affect our overall findings.* ***“***
